# Signalling pathways in hepatocellular carcinoma (HCC) metastasis and invasion: Molecular mechanisms and therapeutic implications

**DOI:** 10.1016/j.bbrep.2025.102403

**Published:** 2025-12-09

**Authors:** Jayanta Das, Bhupen Barman, Phulen Sarma, Bipul Kumar Das, Rajiv Chetia, Partha Pratim Kalita

**Affiliations:** a Department of Biochemistry, All India Institute of Medical Sciences, Guwahati, 781101, India; b Department of Medicine, All India Institute of Medical Sciences, Guwahati, 781101, India; c Department of Pharmacology, All India Institute of Medical Sciences, Guwahati, 781101, India; d Department of Pediatrics, All India Institute of Medical Sciences, Guwahati, 781101, India; e Animal Health Laboratory, ICAR- National Research Centre on Pig, Rani, Guwahati, 781015, India; f Program of Biotechnology, Assam Down Town University, Guwahati, Assam, 781026, India

**Keywords:** Hepatocellular carcinoma, Invasion, Metastasis, Signalling pathways, Tumour microenvironment, Targeted therapy

## Abstract

HCC is one of the deadliest malignancies with a rising global occurrence and poor prognosis. Metastasis and invasion are essential processes in the HCC progression, and have a profound bearing on clinical outcome. This review explores the key signalling pathways involved in HCC metastasis and invasion, focusing on their molecular mechanisms, crosstalk, and therapeutic implications. Alongside the discussion of the Wnt/β-catenin, TGF-β, PI3K/AKT/mTOR, MAPK/ERK, HGF/c-MET, Notch and Hippo-YAP/TAZ pathways, are known to contribute to promoting aggressive HCC behaviour. Stromal interactions, extracellular matrix remodelling, hypoxia and angiogenesis as well as the tumour microenvironment are also highlighted. These pathways are subject to current therapeutic treatments in the form of tyrosine kinase inhibitors and monoclonal antibodies, and research prospective of the Wnt/β-catenin blocker, TGF-β inhibitors, etc. The variations in tumours and resistance patterns to treatment and their existing problems in treating HCC are addressed. The review evaluates new therapeutic targets offering a foundation for further research and clinical advancements in this challenging field.

## Highlights

• Maps key pathways driving metastasis in hepatocellular carcinoma.
• Identifies signalling and immune-evasion mechanisms as therapeutic targets.
• Highlights microenvironment remodelling linked to HCC progression.
• Supports development of biomarkers for early and personalized diagnosis.

## 1 Introduction

### 1.1 Epidemiology and clinical burden of HCC

HCC is the most common primary liver cancer, as well as an important cause of cancer death worldwide with approximately 906,000 new cases and 830,000 annual deaths in 2020 [1]. Geographic variability in the global burden of HCC is influenced by the observed highest incidence rates in Asia and sub-Saharan Africa as a consequence of the high frequency of chronic hepatitis B virus (HBV) infection [2]. On the other hand, the number of HCC cases is increasing in Western countries due to HCV infection; alcohol-related liver disease, non-alcoholic fatty liver disease (NAFLD), and metabolic syndrome as major rising drivers [[3], [4], [5], [6]]. Thus, the major risk factors for developing HCC include chronic viral hepatitis (HBV and HCV), cirrhosis, alcohol, aflatoxin, and metabolic disorders including obesity and diabetes [7,8]. Even with increased surveillance and improved therapeutic strategies, HCC has a poor overall prognosis owing to its high metastatic/invasive potential [2,9,10]. HCC has an etiology-dependent molecular profile that is distinct in HBV-associated and NASH induced subtypes. Actionable mutations are only found in 25 % of HCC cases whereas about 50 % of HCC cases have recurrent non-targetable mutations (TP53, CTNNB1, TERT). Despite the development of TERT promoter and WNT/b-catenin pathway inhibitors, the treatment effect has yet to be achieved. The majority of HCC mutations do not have good pharmaceutical targeting therapies [11]. The knowledge of the downstream signalling pathways pertaining to these mutations is important in designing new therapeutics to counteract the undruggable targets and enhancing better response to HCC therapy among different etiological contexts. For instance, despite all the efforts made in recent decades, the 5-year survival rate for metastatic HCC is still under 10 % and thus strongly underlines the necessity to understand molecular mechanisms responsible for its aggressiveness [12]. This article addresses the current status, progress, and problems of the HCC clinical treatment on the background of the molecular genetic changes of HCC. This review further includes a summary of the up to date knowledge about the function and involvement of the HCC signalling pathways involved in HCC metastasis and invasion, their clinical relevance as well as a new therapeutic approach. In order to provide the foundation for development of precision therapies to defeat the lethal consequence of metastatic HCC, we synthesize current evidences about these molecular mechanisms.

### 1.2 Metastasis and invasion: hallmarks of HCC progression

HCC progression can be considered as two critical determinants that are metastasis and invasion, which subsequently affect patient survival and treatment outcomes. HCC with a high potential of metastasis, and extrahepatic metastases are found in about 30–50 % of the patients [[13], [14], [15]]. The lungs constitute the most frequent extrahepatic site of spread with 43.8–77.8 % of all extrahepatic cases of metastasis and 6.28 % of patients at diagnosis with lung metastasis [[16], [17], [18], [19]]. The second most common site is bone, which happens in 16–52.5 % of cases, with the most common being the vertebral involvement [20,21]. Autopsy cases of lymph node metastasis are found in 17.5–26 % with the abdominal regional nodes mostly affected [22]. In 3.5–10 % of cases, adrenal glands are involved and in 5–20 % of advanced HCC, peritoneal metastasis can be observed [20,23]. The less popular locations are brain (2.5–3.5 %), soft tissues (21.8 %), and the visceral organs [21]. The metastatic spread pattern has a strong impact on prognosis and choice of treatment.

At the time of diagnosis of HCC at an advanced phase with metastases, therapeutic options are very limited and rely on systemic treatment and palliative care. The treatment strategies are additionally complicated by the physiology of the liver and frequent underlying chronic liver disease. While there is the development of targeted therapies and immunotherapy, these treatments have different effects on and are poorly tolerated in patients with impaired liver function [24]. Moreover, heterogeneous nature of HCC and its tendency for intrahepatic spread make disease eradication challenging, even with advanced treatment modalities. To address these challenges, a multi-faceted approach is needed, including continuing research in novel therapeutic approaches and improved early detection and intervention [3]. Tumour progression (dissemination, invasion, and metastasis) is under control of interplay between genetic, epigenetic and microenvironment factors [25,26]. However, current therapies fail to control tumour dissemination, and over 70 % of the patients will develop metastatic recurrence within 5 years following curative resection [27]. Mittal et al. [28] explained the HCC metastasis occurs through dysregulation of major signalling pathways of EMT, ECM remodelling and angiogenesis, which promote motility, survival and colonization of tumour cells [28].

A number of signalling pathways along with alterations in cellular metabolism and metabolic plasticity play the central roles in HCC metastasis and invasion [29,30]. Tumour EMT is regulated by genes frequently regulated by hyperactivated Wnt/β-catenin pathway that is frequently hyperactivated in HCC. Activation or mutation of oncogenes in this pathway causes the loss of E-cadherin and expression of the mesenchymal markers -vimentin (Craig et al., 2020). Similarly, the TGF-β pathway converts from a tumoural inhibiting to a pro-metastatic role in advanced HCC, which triggers immunosuppresion and ECM degradation by matrix metalloproteinases (MMPs) [31]. HGF/cMET pathway activates cytoskeletal reorganization and invasion; PI3K/AKT/mTOR is involved in tumour cell survival and chemoresistance [32]. Further, the tumour microenvironment (TME) is involved in remodelling the distant site for colonization as hepatic stellate cells, cancer-associated fibroblasts (CAFs) and exosome mediated signalling pathways are used to precondition the distant site for colonization [[33], [34], [35]].

### 1.3 Significance of signalling pathways in HCC

Despite progress in the development of targeted therapies, existing methods are intrinsically limited by issues such as intratumoural heterogeneity, redundant signalling pathways, and adaptive resistance [12]. Treatment resistance may be overcome through inhibition of Wnt/β-catenin and TGF-β signalling, novel targeted nanotherapeutic approach, and various combination strategies such as immune checkpoint blockade and the inhibition of TGF-β.

## 2 Key signalling pathways in HCC metastasis and invasion

**Fig. 1:**
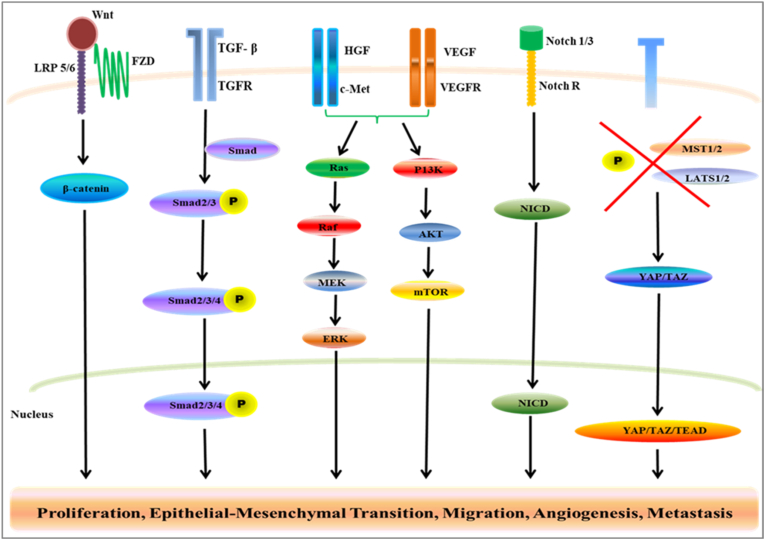
Overview of signalling pathways in HCC metastasis and invasion. An illustration of the HCC signalling pathways and their significant constituents related to the metastasis and invasion, including signalling pathways, viz., Wnt-β-catenin, TGF- β, P13K/AKT/mTOR, MAPK/ERK, HGF/C-Met, Hippo, and Notch signalling pathway.

The processes of metastasis of HCC involves many steps such as the detachment of tumour cells from a tumour, entry into circulation, survival in circulation, and finally implant in distal organs. Disruption of signalling pathways (including epithelial-mesenchymal transition (EMT) program/mechanism, intravasation and extravasation through changes in the extracellular matrix (ECM), angiogenesis, and immune evasion) then facilitates these stages. This chapter focuses on the critical pathways involved in HCC metastasis and the molecular mechanism for these pathways, the interaction that occurs among these identified pathways, and the new therapeutic strategies for treating HCC. The essential overview of signalling pathways to govern HCC invasion and metastasis is shown in Fig. 1.

### 2.1 Wnt/β-catenin pathway

#### 2.1.1 Activation of the Wnt/β-catenin pathway in HCC

Genetic and epigenetic modifications of HCC lead to abnormal activation of Wnt/β-catenin signalling pathway. However, other 30–40 % of HCC cases have CTNNB1 (β-catenin) mutations or inactivating mutations of AXIN1 or APC. Thus, these mutations immerge with the β-catenin degron complex (APC/Axin/GSK3β/CK1α) that shuts off degradation of β-catenin and the β-catenin accumulate in the cytoplasm (Craig et al., 2019; [36,37]). Activation of the pathway is mediated by Wnt ligands (Wnt1 and Wnt3a) secreted by HCC cells and tumour associated macrophages. Therefore, these ligands bind to FZD receptors and LRP 5/6 co-receptors that leads to the phosphorylation of DVL and subsequent translation of further signalling [38,39]. Moreover, further environmental factors such as chronic HBV/HCV infection, hypoxia, and SFRP1 (and other antagonists) promoter methylation result in the activation of pathway through Wnt receptor (including overexpressing Wnt ligand) [[39], [40], [41]]. These stabilize β-catenin so that it can translocate to the nucleus to carry out their roles in transcription.

#### 2.1.2 Molecular mechanisms of β-catenin signalling

In the absence of Wnts, β-catenin is kept in a destruction complex (Axin/APC/GSK3β/CK1α) and is sequentially phosphorylated by CK1α and GSK3β to mark it for ubiquitination and subsequent proteasomal degradation [42,43]. Activation of the pathway using phosphorylation of LRP5/6 thus disrupts this complex, allowing β-catenin to accumulate and translocate to the nucleus. In the nucleus, under the stimulation of Wnt signalling, β-catenin binds to TCF/LEF transcription factors and co-activators (CBP/p300) to promote the expression of oncogenic targets such as c-MYC and Cyclin D1, and upon transcriptional activation, lead to β-catenin accumulation [44,45]. In HCC, β-catenin further downregulates E-cadherin (an inhibitory factor of β-catenin), upregulates N-cadherin and vimentin (pro LGR5 and CD44 also maintain cancer stem cell (CSC) population and resulting in chemoresistance and recurrence [46].

#### 2.1.3 Crosstalk with oncogenic pathways

TGF -β signalling in combination with Wnt/β-catenin signalling accelerates the development of HCC by increasing expression of matrix metalloproteinases (MMPs) induced by TGF -β, which promote ECM degradation and vascular invasion [47]. Metabolic stress stabilizes β-catenin through hypoxia inducible factors (HIF1α) and hepatocyte growth factor (HGF) [[48], [49], [50]]. One of the non-genetic β-catenin reinforcement pathways which facilitates nuclear retention and transcriptional activity of β -catenin thus contributing to early recurrence of HCC is the AKIP1/β -catenin/CBP axis [51]. TMEM9 interacts with v-ATPase, promotes APC degradation at lysosome, which is independent of genetic mutations to activate β-catenin and result in downstream hepatic tumourigenesis ([52]; Xue et al., 2019). Furthermore, Wnt/β-catenin signalling stimulates inflammatory pathways through WNT7B ligand from macrophages resulting in pro-tumour microenvironment formation in cholangiocarcinoma, [53]. These interactions reveal that the pathway acts as a feed forward loop to promote HCC metastasis and resistance to therapeutic interventions by integrating stroma, metabolic, and inflammatory signals.

### 2.2 TGF-β pathway

#### 2.2.1 Activation of TGF-β pathway in HCC

The ligand and receptor, as well as the environment derived from the stroma, can activate the TGF-β signalling pathway in HCC. However, cancer associated fibroblasts (CAFs) and tumour associated macrophages (TAMs) in TME are the main sources for such secretion locally [54]. However, in contrast, TGFβ binds heterodimer transmembrane serine/threonine kinase receptors TGFβRII and TGFβRI with the phosphorylation of SMAD2/3 in cytosol (ligand--TGFβRII-- recruits/phosphorylates TGFβRI--SMAD2/3 phosphorylation). The phosphorylated SMADs (together) form a complex with SMAD4 and translocated into the nuclear region to regulate the transcription [[55], [56], [57]]. Moreover, an increase in TGF-β receptor further contributes to TGF-β transduction in cellular TGF-β signalling caused by environmental TGF-β contributors (i.e., chronic inflammation, fibrosis, and NAFLD induced metabolic stress) [11,58,59]. The overexpression of TGF-β has been proven to participate in cirrhosis, in the conversion of hepatic progenitor cells into hepatoma initiating cells [60,61] to maintain the carcinogenic processes [62].

#### 2.2.2 Molecular mechanisms of TGF-β pathway

The TGF-β signalling pathway functions through both SMAD-dependent and SMAD-independent mechanisms. Upregulation of SNAIL and TWIST, downregulation of E-cadherin, and upregulation of vimentin by canonical SMAD signalling pathway promotes the EMT, resulting in the metastasis [31,[63], [64], [65]]. In addition to the conventional pathways, PI3K/AKT/mTOR and MAPK/ERK pathways, TGF-β signalling allows cell survival, chemoresistance, and proliferation. In particular, PI3K acts on PIP2 to produce PIP3, which activates AKT/mTOR and stimulates anabolism and cell cycle transition, and RAS/RAF/MEK/ERK signalling promotes angiogenesis and differentiation [37,[66], [67], [68]]. TGF-β also supports cancer stem cells (CSCs) by promoting the expression of CD44 and CD133 and suppresses MHC-I/II and recruits Tregs/MDSCs [31,58,69].

#### 2.2.3 Crosstalk with other pathways

HCC progression is promoted through TGF-β working in combination with oncogenic pathways and stromal components. Furthermore, it interacts with Wnt/β-catenin signalling and with epidermal growth factor (EGF) signalling to enhance epithelial-mesenchymal transition (EMT) and maintenance of cancer stem cells (CSC) [[70], [71], [72], [73]]. TGF-β derived from hepatic stellate cells (HSC) and TGF-β upregulates the metabolic enzyme, NNMT, within the tumour microenvironment (TME) [74], which is implicated in poor prognosis, and induces fibrosis through collagen production (Pellicoro et al., 2014). TGF-β′s tumour suppressing effects are abrogated by interaction with inflammatory pathways including platelet derived growth factor (PDGF), and EGF autocrine loops, which enable immune evasion and metastatic spread [75]. Galunisertib (LY2157299) is a TGF-β inhibitor that crosses the blood brain barrier and therefore inhibits both SMAD and non-SMAD pathways in a clinical setting, results in reduced EMT and metastatic burden. Sorafenib in combination therapy or treatment with PD-L1 inhibitors (for example, atezolizumab) increased therapeutic efficacy by overcoming immune suppression [[76], [77], [78], [79], [80]]. Chimeric antigen receptor T (CAR-T) cells (such as PSMA–TGFβRDN) have been developed as an evolving strategy but trials of HCC are yet to come [81].

### 2.3 PI3K/AKT/mTOR pathway

#### 2.3.1 The PI3K/AKT/mTOR pathway activation in HCC

Hyperactivation of the PI3K, AKT and mTOR pathway is not uncommon in HCC as a result of genetic alteration or at the level of an upstream signalling event. In this hyperactivation, PTEN, a tumour suppressor, commonly has loss-of-function mutations, and PIK3CA, encoding the catalytic subunit of PI3K, has gain-of-function mutations, leading to unchecked endpoints of PIP3 and then AKT activation [[82], [83], [84]]. After ligand binding to receptor tyrosine kinases (RTKs), including EGFR and IGF-1R, PI3K is activated catalyzing the transformation of PIP2 to PIP3 and its recruitment of AKT to plasma membrane to be phosphorylated by PDK1 and mTORC2 [43]. This signalling cascade is further amplified by growth factors such as EGF and IGF, as well as oncogenic RAS. For example, by failing to degrade the product PIP3, the loss or epigenetic silencing of PTEN, the pathway is exacerbated in hyperactivity [70,85]. Long non-coding RNAs (lncRNAs) including RHPN1-AS1 and PICSAR, in addition facilitate the activation of the PI3K/AKT/mTOR pathway through binding to tumour repressive miRNAs, e.g miR-7-5p or through upregulation of pathway components [86,87].

#### 2.3.2 Molecular mechanisms of PI3K/AKT/mTOR pathway

The downstream effectors of the PI3K/AKT/mTOR signalling pathway drive oncogenesis by controlling cellular survival, proliferation and metabolism. AKT also phosphorylates pro-apoptotic proteins [88,89], allowing it to inhibit apoptosis, and also stimulates mTORC1, which in turn activates HIF-1α to promote angiogenesis and S6K/4E-BP1 to augment protein synthesis. Moreover, mTORC2 phosphorylates AKT doing establishing a feed forward loop ensuring pathway activity and inducing cytoskeletal remodelling [90]. In HCC, mTORC1-dependent lipid and nucleotide synthesis is required to support rapid tumour growth. PIP3-mediated AKT signalling is exacerbated in the context of PTEN deficiency contributes to chemoresistance [34,35,91]. Furthermore, dysregulation of E2F transcription factors, specifically E2F1 further contributes to tumour progression, activates the PI3K/AKT/mTOR pathway and inhibits apoptosis [71,[92], [93], [94], [95]]. HCC subclasses are found to be characterized by pathway upregulation of downstream targets SYK, RHEB and pS6K, which are biomarkers for aggressive disease [96].

#### 2.3.3 Crosstalk with other signalling pathways

RAS directly activates PI3K and feeds back to inhibit insulin receptor signalling via mTORC1-S6K1, which exacerbates rewiring of metabolic signalling [94,[97], [98], [99], [100]]. Hypoxia induced HIF-2α works in concert with PI3K/AKT/mTOR to upregulate lipid synthesis to advance steatotic HCC [101]. Autophagic signalling is connected to drug resistance; SOCS5 suppresses PI3K/AKT/mTOR-dependent autophagy that, in turn, enhances HCC metastasis. PTEN is lost (that synergizes with NF-kβ) to maintain inflammation driven tumourigenesis [94,95,102,103]. PI3K/mTOR inhibitors such as copanlisib and everolimus are clinically tested in combination with sorafenib or immunotherapy (NCT03735628; NCT01239355), which still suffer from resistance due to other pathway compensation including IGF-1R/AKT reactivation [74,104]. Third generation mTOR inhibitors like rapalink aim to get over the resistant by attacking both mTORC1/2 and upstream feedback loops simultaneously [105].

### 2.4 MAPK/ERK pathway

#### 2.4.1 The MAPK/ERK pathway activation in HCC

Genetic mutations and upstream receptor signalling of the MAPK/ERK signalling pathway is often hyperactivated in HCC. HCC occurs in 5–10 % of subjects due to activating mutations of RAS genes (e.g., HRAS and KRAS), or of BRAF, which causes pathway activation constitutively [67,96]. RAS activation occurs in response to ligand binding to receptor tyrosine kinases (RTKs), which includes EGFR, VEGFR, and IGFR, by means of the Grb2/Shc/SOS adaptor proteins that act to convert RAS to its GTP bound state and activate the RAF/MEK/ERK cascade [106,107]. Loss of RKIP interferes in Raf-MEK interaction, and loss of DUSP1 eliminates ERK feedback inhibition [108]. Along with RAS activation, decreased expression of Sprouty2 (SPRY2) and hypermethylation of promoter of RasGAPs (DAB2IP and RASAL1) correlates with stages of HCC progression and recurrence [67,109,110]. Moreover, the interaction of HBV in the host DNA activates the MAPK/ERK pathway through transcriptional activators, involving viral infection to oncogenic signalling [111].

#### 2.4.2 Molecular mechanisms of MAPK/ERK pathway

Successive phosphorylation of RAF, MEK and ERK kinases reflects the MAPK/ERK signalling cascade. Upon activation ERK translocates to the nucleus phosphorylating transcription factors, ELK1, c-Jun and c-Fos, among others. The phosphorylation of this event results in an upregulation of MMP2/9 which degrade the extracellular matrix (ECM), and facilitate metastasis [67,112]. In addition, in effecting cell cycle progression by Cyclin D1 and blocking the trigger of apoptosis, the ERK-driven AP-1 family of transcription factors also promotes tumour survival [113,114]. The activation of MEK1/2 and ERK is known in HCC samples, where MEK1/2 is hyperphosphorylated in 1,4 times higher levels than in non-tumour tissues and the ERK activation is linked to a poor prognosis [67]. As a result, ErbB3 phosphorylation is elevated in the pre-treated clinical model and we observe compensatory MEK phosphorylation and ERK reactivation, which is temporarily repressed by a RAF inhibitor, sorafenib, but occurs through resistance via ERK activation [115,116].

#### 2.4.3 Crosstalk with pathways

The interaction of MAPK/ERK signalling pathway with NF-κB increases the production of pro inflammatory cytokines, like IL-6 to support the survival of tumour cells from circulation [113,117]. Furthermore, RAS binds to PI3K comprising cell proliferation and metabolic reprogramming crosstalking the PI3K/AKT/mTOR pathway [107,118]. The synergistic effect between MAPK/ERK, the HBV induced signalling and hypoxia also plays a role in angiogenesis and epithelial mesenchymal transition (EMT) [67]. However, MEK inhibitors (such as trametinib), with sorafenib and immunotherapy are given to counter resistance and clinical trials (e.g. NCT02292173) had shown to be ineffective of pathways redundancy [115,119]. Targeting both the MAPK/ERK and PI3K/AKT/mTOR pathways will address compensatory mechanisms since synergistic inhibition of tumour growth has been demonstrated in preclinical models of CI-1040 and sorafenib [119].

### 2.5 HGF/c-MET axis

#### 2.5.1 The HGF/c-MET pathway activation in HCC

The HGF/cMET pathway is activated in a ligand receptor interactions or a genetic alteration manner. Hepatic stellate cells (HSCs), endothelial cells and Kupffer cells secrete hepatocyte growth factor (HGF) that binds c-MET receptor leading to its dimerization and autophosphorylation of tyrosine residues [120,121]. Activation of HGF by canonical activation results in the phosphorylation of the cytoplasmic domain of cMET which in turn leads to the activation of signalling pathways like Ras/Raf/MEK/ERK and PI3K/AKT [122]. Activation also occurs through hypoxia, EGFR crosstalk or the HGF analog des-γ-carboxy prothrombin (DCP), all of which bypass ligand requirement (eg., ligand-independent activation) [123]. Constitutive signalling and carcinogenesis ([124]; Xue et al., 2024) follow from genetic aberrations: OXR1-aMET gene rearrangements and mutations in the c-MET Y1003 domain that impede ubiquitination and degradation; overexpression of c-MET is present in 20–50 % of HCCs and links with high disease stage and poor prognosis [125].

#### 2.5.2 Molecular mechanisms

Downstream effectors of HGF/c-MET signalling regulate proliferation, survival and metastasis and hence facilitate tumour progression. There is recruitment of adaptor proteins like GRB2 and GAB1 as well as activation of SRC kinase to stimulate cytoskeletal rearrangement via RAC1/CDC42 or formation of invadopodia that leads to extracellular matrix (ECM) proteolysis [126,127]. Therefore, the following key pathways involved are PI3K/AKT/mTOR, promoting cell survival, MAPK/ERK, mediating cell proliferation and STAT3/NF-κB, driving inflammation and angiogenesis [128]. c MET upregulates VEGFA, under hypoxic conditions via hypoxia inducible factor-1 (HIF-1), which promotes angiogenesis necessary for tumour growth [128,129]. LINC00240 and miR-101 modulate this pathway through HGF/c-MET or its downstream effectors, which affect tumour cell viability and invasion [43,130].

#### 2.5.3 Crosstalk with oncogenic pathways

An oncogenic axis of the HGF/c-MET interacts aggressively with oncogenic pathways cooperating in a synergistic approach to promote the aggressiveness of HCC. The metastatic potential is coactivated with epidermal growth factor receptor (EGFR) or integrins and augments metastatic potential through shared downstream effectors such as MAPK and PI3K [131]. Receptors such as RON and IGFR-1 signal under ligand limited conditions by transactivating [132]. NF-κB and STAT3 pathways crosstalk with enhance the production of pro-inflammatory cytokines like IL-6, which create a tumour permissive microenvironment. The state of metabolic stress in hypoxia stabilizes c-MET expression and thus links metabolic stress to angiogenesis and the response to therapy [133]. Despite significant clinical significance in patients with high c-MET expression, c-MET inhibitors, such as cabozantinib, are soon followed by resistance through compensatory HGF autocrine loops, especially after treatment with sorafenib [134].

### 2.6 Notch signalling

#### 2.6.1 The Notch signalling pathway activation in HCC

The ligand-receptor interactions between neighbouring cells activate the Notch signalling pathway, when macrophages or stromal cells express ligands; DLL1, DLL4, and Jagged1/2, which will bind to Notch receptors (Notch 1–4) on hepatocytes or HCC cells [82,135]. This occurs upon binding of ligands, in which ADAM proteases and γ secretase mediate the proteolytic cleavage of Notch receptor and release of Notch intracellular domain (NICD) that translocates to the nucleus [[93], [94], [95],^136^]. Canonical pathways that depend on ligand interaction are also associated with factors that include hypoxia, HBV-associated HBx protein (via the HBx-DLL4-Notch1 axis) or crosstalk with receptors [137,138]. Notch1, Notch4 and ligands (DLL4, JAG1) are overexpressed in >70 % of HCC cases and related to advanced tumour stage and vascular invasion [137]. For example, RO4929097, a gamma-secretase inhibitors inhibit NICD generation and suppress cancer stem cells-driven metastasis [139,140].

#### 2.6.2 Molecular mechanisms of Notch signalling pathway

Nuclear NICD binds with CSL transcription factors like RBPJ-κ to activate target genes as HES1, HEY1, cMyc and cyclin D1. The expression of these genes inhibits differentiation, maintenance of CSCs, and induction of chemoresistance [82,141]. The upregulation of VEGF-A by Notch signalling is coupled with its role in angiogenesis, and it cooperates with the Hedgehog pathway to maintain stable tumour vessels [[93], [94], [95]]. The HBx protein also promotes survival of tumour through activation of DLL4-Notch1 in the context of HBV-related hepatocellular carcinoma [138]. Notch1 and Notch4 overexpression are related to a poor prognosis and LINC00261/MCM6 affect proliferation through Notch-dependent pathways [43]. Moreover, the disruption of regulation of Notch3 and Jagged1 is found to enhance aggressiveness and metastatic potential of HCC [43].

#### 2.6.3 Crosstalk with pathways

Progression of HCC is synergistically facilitated by interactions of Notch signalling with several pathways. Notch signalling engages with the Wnt and Hedgehog pathways to maintain CSCs, the PI3K/mTOR pathway to promote cell proliferation, as well as the VEGF pathway to induce angiogenesis (Jeng et al., 2023). The induction of proinflammatory cytokines release from TAMs following Notch activation by DLL4 also results in an immunosuppressive microenvironment [142]. On the other hand, the interaction with Hippo/YAP pathway forms a feedback loop where the YAP/TAZ activation potentiates Notch signalling and accelerates tumourigenesis [[143], [144], [145], [146]]. Notch and β-catenin pathways are interdependent. DLL4 inhibition leads to a decrease in angiogenesis, while Jagged1 augments vascular invasion by increasing the activity of VEGF; these mechanisms consequently link Notch signalling to vascular invasion [133].

### 2.7 Hippo-YAP/TAZ pathway

#### 2.7.1 The Hippo-YAP/TAZ signalling pathway activation in HCC

In HCC, the Hippo-YAP/TAZ signalling pathway is rendered inactive due to disruptions in its core kinase cascade, resulting in the nuclear translocation of YAP/TAZ. In the normal physiological conditions, upstream kinases MST1/2 and LATS1/2 are activated by density and mechanical stress, and in turn phosphorylate YAP/TAZ and retain them in the cytoplasm or degrade them [147,148]. Disruption of this cascade is observed in the context of HCC whenever genetic or epigenetic modification of MST1/2, LATS1/2 or tumour suppressors, such as NF2 and KIBRA, occurs. Thereby, dephoshporylated YAP and TAZ can skip 14-3-3 protein binding and translocate to the nucleus [149,150]. Furthermore, growth factors such as Wnt, EGF, SHH, and hypoxic conditions independent of EMT activators, that are also capable of agonizing YAP/TAZ and consequently exacerbate dysregulation of the pathway [151].

#### 2.7.2 Molecular mechanisms of Hippo-YAP/TAZ signalling

TEAD transcription factors interact with nuclear YAP/TAZ complexes and lead to the expression of oncogenic genes like CTGF, BIRC5 (survivin), CYR61 that are implicated in driving cellular proliferation, inhibition of apoptosis and chemoresistance [152]. In addition, YAP/TAZ enables extracellular matrix (ECM) remodelling via LOXL2, where LOXL2 mediated stiffening promotes invasion for metastasis [153]. Further, the clinical correlation reveals that YAP is overexpressed in advanced stages of hepatocellular carcinoma (HCC), with vascular invasion, in the prognosis and is expressed in 54 % of HCC tissues with nuclear YAP, as opposed to very weak staining in normal hepatic tissues [154,155]. YAP has been shown to be tumourigenic in transgenic mouse models that corroborate this function as YAP overexpression alone is sufficient to induce HCC [155].

#### 2.7.3 Crosstalk with oncogenic signalling

The Hippo-YAP/TAZ signalling pathway intersects with various oncogenic networks. Synergism of YAP/TAZ with Wnt/β-catenin and Notch pathways promotes the maintenance of cancer stem cells (CSCs) and EMT, and collaboration with STAT3 potentiates proinflammatory cytokines, like IL-6 [143]. YAP functions together with HIF-1α to increase the expression of glycolytic enzymes, such as PKM2, in hypoxic microenvironments to shuttle flux to cell proliferation [151,156]. Moreover, YAP/TAZ activation in CCA is known to promote angiogenesis and proliferation through transcriptional activation of VEGF and TEADs [157]. LOXL2 mediated stiffening of the extracellular matrix (ECM) provides for feedback loops that perpetuate mechanical signalling to maintain YAP/TAZ activity and creates a pro-tumourigenic niche [158].

## 3 Tumour microenvironment (TME) and metastatic niche

**Fig. 2:**
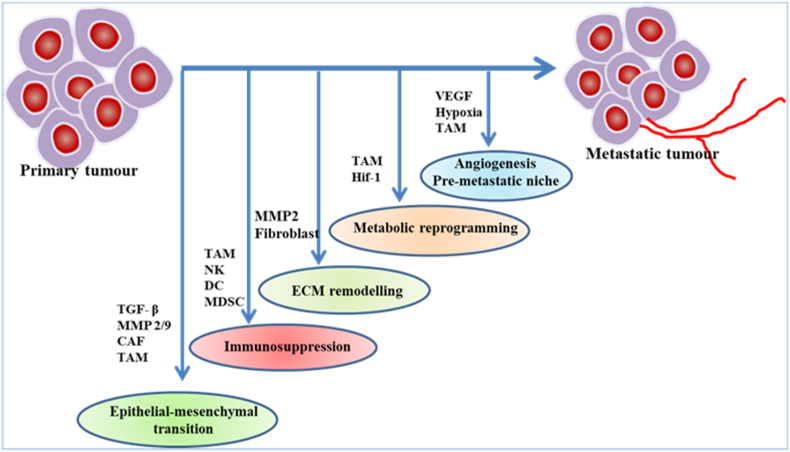
TME metastatic niche formation of Hepatocellular carcinoma (HCC).

The tumour microenvironment (TME) in HCC constitutes an intricate network of cellular and non-cellular components that collectively aid tumour progression, metastasis, and immune evasion. The TME includes tumour cells, stromal cells (for example, activated hepatic stellate cell (HSC), cancer–associated fibroblast (CAF), tumour associated macrophage (TAM), myeloid-derived suppressor cell (MDSC), tumour associated neutrophil (TAN), endothelial cell, and mesenchymal stem cell (MSC)), extracellular matrix (ECM) proteins, digestive enzymes, cytokines, and growth factor and extracellular vesicles as well as exosomes [159,160]. Hypoxia and immune components shape the TME and further influence to promote a pro tumourigenic niche [161,162]. The schematic representation of TME-metastatic niche of HCC are demonstrated in Fig. 2.

### 3.1 Cancer-associated fibroblasts (CAFs)

Cancer associated fibroblasts (CAFs) are the most common cell type in the hepatocellular carcinoma (HCC) tumour environment and significantly contribute to tumour progression and metastasis. CAF is derived from the resident fibroblasts, stellate cells and other precursors also which are activated by signal from tumour cells and stroma [[163], [164], [165]]. Besides providing therapeutic resistance [166,167], they secrete various growth factors, cytokines, and extracellular matrix proteins that help tumour proliferation, angiogenesis, and immune suppression. IL6/IL6R/STAT3 and HIF1α/ZEB1 pathways [168] induce epithelial to mesenchymal transition (EMT) [169] and CAFs secrete EGF, FGF and HGF for tumour growth [170]. In addition, they inhibit apoptosis by the SDF-1/CXCR4 axis, and promote cancer stemness by IL-6/STAT3/Notch signalling [171,172]. CAFs facilitate immune evasion by inducing myeloid-derived suppressor cells and modulating immune cell functions [173]. Their exosomes, with specific miRNAs and lncRNAs, further promote metastasis [[144], [145], [146],^174^]. CAFs also contribute to the development of pre-metastatic niches, thereby reinforcing tumour aggressiveness [175]. In addition, CAFs mediate direct cell-to-cell interaction mainly with immune and endothelial cells to amplify pro tumourigenic signalling, thus creating a supporting niche for tumour cell dissemination. By playing these multifaceted functions, CAFs have an important role in driving HCC metastasis, and thus, they may be potential targets for novel therapeutic interventions.

### 3.2 Immune cells in TME

In hepatocellular carcinoma (HCC), the tumour immune microenvironment is highly dysregulated and can support immune evasion as well as metastasis. It involves contribution from various immune cells, such as tumour associated macrophages (TAMs), myeloid derived suppressor cells (MDSCs) and regulatory T cells (Tregs). In HCC, TAMs most often display M2 like phenotype, producing anti-inflammatory cytokines including IL 10 and TGF β, which facilitate angiogenesis, tissue remodelling, and immune suppression [94,95,102]. Furthermore, TAMs have the expression of programmed death-ligand 1 (PD-L1) that suppresses T cell activation and also enhances immune evasion [176]. Lastly, the MDSCs, or immature myeloid cells are a heterogeneous population of cells that suppress T cell responses through the production of arginase-1 and inducible nitric oxide synthase (iNOS), resulting in nutrient depletion and ROS generation [177]. Accumulation of Tregs, a subset of CD4^+^ T cells, within the tumour microenvironment decreases the function of effector T cells, and prevents effective anti-tumour T cell responses. Additionally, the interaction between these immune cells with expression of immune checkpoints PD-1 and CTLA-4 results in immune malfunction, causing angiogenesis, extracellular matrix remodelling and metastasis [16,17,178]. There is a close connection between this immunosuppressive network and poor prognosis in HCC patients.

### 3.3 Tumour-associated macrophages (TAMs)

Tumour associated macrophages (TAMs) within the hepatocellular carcinoma (HCC) tumour microenvironment are important immune cells that are polarized to either tumour suppressive or tumour promotional functions. Local cytokines can influence TAMs to differentiate into M1 macrophages, high level of antigen presenting and high level of production for pro-inflammatory cytokines such as IFN-γ and reactive oxygen species (ROS), or M2 macrophages which is induced by Th2 cytokines (IL-4/IL-13) and TGF-β low level of antigen presenting, immunosuppressing [35,[179], [180], [181], [182]]. It has been shown that macrophages in HCC skew towards M2 phenotype and HCC with high M2 TAMs is associated with aggressive phenotype and poor prognosis [183,184]. Various processes that are mechanistically linked together allow TAMs to promote the metastasis of HCC. They secrete pro-angiogenic factors, as VEGF, EGF, PDGF, TGF-β, that initiate neovascularization, as well as matrix metalloproteinases, such as MMP-2, MMP-9, which remodel extracellular matrix in favor of tumour cell invasion [[185], [186], [187]]. Moreover, TAMs take part in epithelial-mesenchymal transition (EMT) by triggering TLR4/STAT3 signalling pathways and releasing cytokines such as IL-8, TNF-α and TGF-β1 as well as proteases including MMP17 [[188], [189], [190]]. Moreover, TAMs accomplish this by promoting immune evasion: generating regulatory T cell infiltration via the production of IL-10 and TGF-β, as well as chemokines [191]; while also expressing immune checkpoint molecules such as PD-1 that interact with PD-L1 ligand on tumour cells impeding the function of CD8^+^ T cells [192,193]. In addition, TAMs are involved in sorafenib resistance in the setting of therapy and lowering their numbers improves therapy outcome [194,195]. These findings together indicate that M2 type TAMs may be a new strategy to impede HCC metastasis and recurrence [196].

### 3.4 Extracellular matrix (ECM)

The extracellular matrix (ECM) represents a complex network of proteoglycans, glycoproteins, and polysaccharides, contributing both structural support and biochemical signalling within the tumour microenvironment. In HCC, the ECM undergoes dynamic modifications that enable tumour growth, invasion, and metastasis [25,197]. Cell adhesion, migration, and signalling pathways involved in tumour progression are deregulated by dysregulation of ECM components including collagens, laminin, and heparan sulfate proteoglycans such as GPC3 ([35,182]; Liu e al., 2018). Matrix metalloproteinases (MMPs) and urokinase-type plasminogen activator (uPA) remodelling enzymes degrade the ECM and basement membrane to promote tumour cell dissemination and metastatic focus establishment [[93], [94], [95],^198^]. Additionally, modified ECM stiffness and composition form a barrier that prevents drug penetration and a part of therapeutic resistance [199]. In short term, HCCs ECM has become altered and provide the physical scaffold for tumour cells, however, it is actively compensating in signalling processes to prime the tumour cells for metastasis [[200], [201], [202]]. Such complex HCC interactions in the ECM and their action contribute to HCC progression and can potentially serve as therapeutic targets.

### 3.5 Hepatic stellate cells (HSCs)

Hepatic stellate cells (HSCs), situated in the space of Disse, exist in both quiescent and activated states. Activation occurs in response to liver injury, resulting in extracellular matrix (ECM) accumulation and fibrosis [203,204]. Activated HSCs (a-HSCs) facilitate hepatocellular carcinoma (HCC) metastasis through various mechanisms. However, paracrine signalling mediates their role in HCC cell proliferation, migration, invasion, as well as enhancing tumour growth, as they activate pathways, including NF-κB, ERK, IL-6/STAT3 and Wnt/β-catenin [205,206]. Furthermore, a-HSCs secrete pro-angiogenic factors, such as VEGF, angiopoietin-1, IL-8, and modify the MMP/TIMP balance in order promote angiogenesis and metastatic microenvironment [207,208]. In addition, HSCs control immunosuppression by interacting with the monocytes, macrophages, and T, and regulatory T cells (Treg) to reduce IFN-γ production, to induce apoptosis via PD-L1, and promote the immunosuppressive state by TGF-β1, COX2-PGE2-EP4 and GARP complex pathways [[209], [210], [211]]. Moreover, HSCs participate in chemoresistance by means of laminin-332/α3 integrin, FAK ubiquitination, and FGF9 secretion [212,213]. The clinical studies associate activity of HSC with HCC recurrence and poor prognosis [94,95,102,214].While some evidence suggests tumour-suppressive roles, such as endosialin-mediated inhibition of HCC proliferation, the predominant view underscores HSCs as facilitators of HCC progression. Bidirectional interactions between HSCs and HCC cells create an immunosuppressive, pro-metastatic niche, positioning HSCs as potential therapeutic targets [94,95,102,214].

### 3.6 Epithelial-mesenchymal transition

Epithelial-mesenchymal transition (EMT) -a dynamic and reversible biological program is pivotal process in the metastasis of HCC, marked by the loss of epithelial markers such as E-cadherin and cytokeratins, and the acquisition of mesenchymal markers like N-cadherin and vimentin, which facilitate tumour cell invasion and dissemination [99,100,[144], [145], [146]]. Disruption of cell-cell adhesion thus, allows tumour cells to penetrate through the basement membrane and spread hematogenously as the circulating tumour cells (CTCs) which are correlated to recurrence and poor prognosis [215]. Transcription factors (TFs) Snail (SNAI1), Slug (SNAI2), ZEB1/2, and members of TWIST family regulate EMT [216,217]. TIP30 deficiency causes the occurrence of nuclear Snail, which leads to E-cadherin repression in HCC through EMT [31]. While, in cooperation with TGF-β, the Axl receptor tyrosine kinase phosphorylates Smad3 which induces Snail and MMP9 upregulation, and increases the invasiveness. HNRNPAB induces EMT through the transactivation of Snail [218,219]. The β-catenin/TCF4 signalling protein complex, which promotes EMT by β-catenin/TCF4 mediated expression of Snail/Slug in response to Wnt signals, is inhibited through a direct binding of HNF4α to TCF4 [220]. In this case, CLDN3 epigenetic silencing triggers EMT as Wnt/β catenin and Slug are activated [221]. Thrombospondin 4 and lysyl oxidase, amongst several other oncogenes, additionally drive EMT associated metastasis [222]. Furthermore, EMT confers stem like properties to HCC cells, including self-renewal and early relapse [223]. Targeting EMT-TFs and downstream pathways, such as AXL and Wnt/β-catenin, may represent therapeutic options for counteracting HCC progression.

### 3.7 Hypoxia and angiogenesis

Hypoxia and angiogenesis are pivotal factors in the metastasis of HCC. Rapid tumour growth and aberrant vascular architecture cause hypoxia (which is the stabilization of hypoxia inducible factors e.g. HIF-1α and HIF-2α). Subsequently, these factors initiate genes for activating angiogenesis, glycolysis, and cell survival [[224], [225], [226]]. During hypoxic conditions in HCC, pro-angiogenic factors, such as VEGF, PDGF, and Ang-2 are secreted triggering the formation of new blood vessels and the recruitment of endothelial cells to the tumour site [226,227]. The resulting neovasculature is abnormal and leaky, but essential nutrients and oxygen are delivered, and tumour cell dissemination and metastasis occurs. In addition, prolonged hypoxia can lead to therapeutic resistance (i.e., sorafenib treatment) can further induce the HIF-1α and NF-κB signalling, promoting a more aggressive and treatment resistant tumour behaviour [228].

#### 3.7.1 Hypoxia-induced molecular signalling pathways

**Fig. 3:**
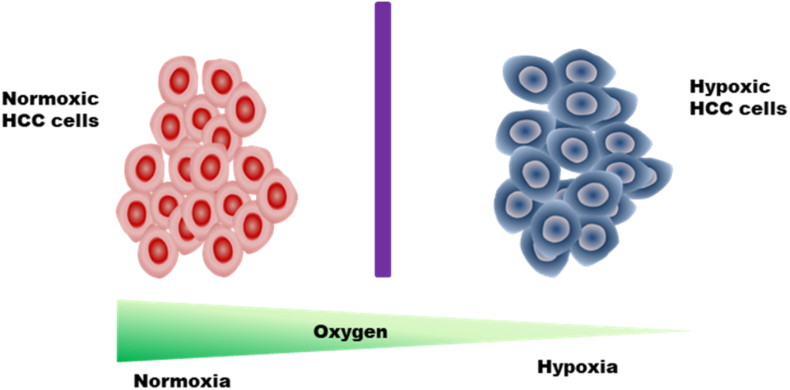
Comparison of Normoxic and Hypoxic TME in HCC. A gradual decrease in oxygen tension or pressure from normoxia to hypoxia condition. Normoxic cells of HCC tumours are more oxygenated due to close propensity of blood vessel whereas hypoxic cells are away from blood vessels result lower oxygen level.

**Fig. 4:**
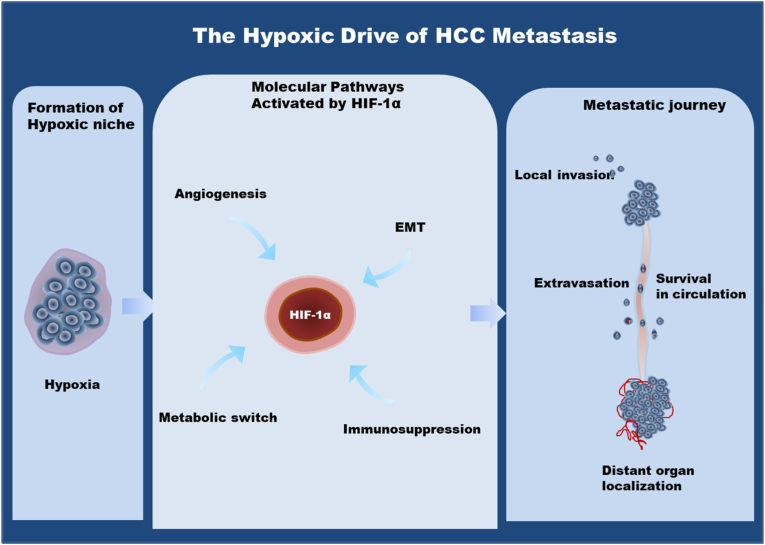
Role of hypoxia in HCC metastasis: From niche formation to organ colonization.

Hypoxia or low oxygen tension is also a characteristic of solid tumours such as HCC due to uncontrolled cell proliferation and aberrant and inefficient vasculature (Fig. 3). This hypoxic microenvironment not just serve as a passive background but drives metastasis by the activation of complex molecular signalling cascades, which is mainly coordinated by the hypoxia-inducible factors (HIFs) or more specifically by the HIF-1α. [229,230]. In normoxic cells, HIF-1α is quickly degraded whereas in the hypoxic niche of an HCC tumour, it is stabilized, dimerizes with HIF-1β and translocates to the nucleus at a slow rate [230,231]. In this case, HIF -1alpha acts as a potent transcriptional regulator by binding hypoxia-responsive elements (HREs) in promoter of more than one hundred downstream targets, and directing a complex pro-metastatic programme [229].The role of hypoxia signalling in HCC metastasis is depicted in Fig. 4.

One of the key routes that are triggered by hypoxic signalling is stimulation of angiogenesis and vascular remodelling. HIF-1α influences the expression of the major angiogenic mediators vascular endothelial growth factor (VEGF) and angiopoietin-2 (Ang-2) directly [231,232]. Whereas VEGF promotes the endothelial growth and development of new vessels, Ang 2 disrupts existing mature vessels. The ensuing synergistic interaction produces an immature and leaky and disorganized vascular network [232]. Paradoxically, this neovasculature often does not alleviate hypoxia, and becomes a self-perpetuating positive feedback loop that however enhances the intravasation of tumour cells to the circulatory system-one of the crucial initial steps in the metastatic cascade [231]. At the same time, hypoxia is a potent stimulus of epithelial-mesenchymal transition (EMT), a developmental mechanism in which the carcinoma cells shed epithelial properties. HCC cells down-regulate cell-adhesion molecules such as E-cadherin and increase mesenchymal markers such as vimentin, through HIF-1α -mediated up-regulation of transcription factors, including Twist, Snail and Slug [231,233]. This transformation reduces intercellular adhesion, enhances migratory ability as well as an invasive phenotype, which allows the separation of cells of the primary tumour mass and invading of adjacent stromal tissue [233].

The process is initiated by the Formation of a Hypoxic Niche within the TME that stabilizes and activates HIF-1 α in the cancer cell. The subsequent activated HIF-1 a then regulates four key molecular programs that are required in metastasis: Angiogenesis, EMT, Metabolic Switch, and Immunosuppression. VEGF stimulated angiogenesis, which facilitates the appearance of new, leaky blood vessels to feed the tumour and escape the body. EMT is also associated with transcriptional factors SNAIL and TWIST that lead to the loss of epithelial properties and the acquisition of mesenchymal invasiveness by cancer cells. Metabolic switch favours the anaerobic glycolysis over the oxidative phosphorylation form of energy production and is marked by an increase in the expression of enzymes such as LDHA and production of lactate enabling survival in low-oxygen conditions. Immunosuppression contributes to the exhaustion or exclusion of cytotoxic T-cells, allowing the tumour to evade immune surveillance. These molecular changes facilitate the processes of Metastatic Journey, which entails the following stages; Local Invasion into the neighbouring tissue, Intravasation into the bloodstream, Survival in Circulation as Circulating Tumour Cells (CTCs), Micrometastasis formation, and eventually, successful Distant Organ Colonization. HIF-1 α is therefore placed at the core of the regulation between the hostile tumour environment and metastatic progression. This figure was created using Microsoft PowerPoint.

This aggressive behaviour is further enhanced by the hypersensitive metabolic reprogramming that is caused by hypoxia. To survive in low oxygen, HCC cells switch their energy synthesis machinery out of efficient mitochondrial oxidative phosphorylation in favor of an anaerobic glycolysis process, the so-called Warburg effect [229]. HIF-1 mediates this metabolic rearrangement by driving the synthesis of glucose transporters GLUT1 and GLUT3 in addition to glycolytic enzymes such as lactate dehydrogenase A (LDHA) and inhibiting mitochondrial activity through pyruvate dehydrogenase kinase 1 (PDK1) [229,230]. The main event of this reprogramming exhibits the increased lactate production and efflux into the tumour microenvironment via monocarboxylate transporter MCT4. This leads to an immunosuppressive environment as well as facilitating invasion and metastasis due to the lactate rich, acidic tumour microenvironment [229,234]. Lactate, along with the adenosine buildup coordinated by hypoxia via the CD39/CD73 pathway, has a direct effect of inhibiting the cytotoxic activity and proliferation of CD8^+^ T cells and natural killer (NK) cells and promoting the growth and polarisation of immunosuppressive cell types such as regulatory T cells (Tregs) and M2-polarised tumour-associated macrophages [229,234]. The immunosuppressive shield allows the circulating tumour cells to escape the immune system, thus increasing their survival in circulation followed by colonization of remote organs.

## 4 Strategies of targeting HCC signalling pathways

HCC metastasis progression requires multiple treatment approaches due to its intricate signal network system. Medical practitioners presently use targeted therapies like tyrosine kinase inhibitors (TKIs) along with monoclonal antibodies for advanced HCC treatment. Practical applications of tyrosine kinase inhibitors (TKIs) continue to serve as the main therapeutic basis but new drug candidates that concentrate on specific pathways demonstrate promising performance in preclinical and clinical contexts. For example, TKIs like sorafenib and lenvatinib have shown efficacy in improving overall survival and progression-free survival by inhibiting multiple kinases involved in tumour growth and angiogenesis. An alternative mechanism of action and a potential for personalized treatment can be offered from monoclonal antibodies as those such as ramucirumab and checkpoint inhibitors. In addition, there is ongoing research into many of the combination therapies, multi-targeting novel targeted therapies and local treatments. There is a move toward identifying the predictive biomarkers and more effective combination strategies of therapy in order to optimize treatment outcomes (to curb side effects, low tolerability and drug resistance) for patients with advanced HCC. Some of the therapeutic agents targeting HCC metastasis signalling pathways are listed in Table 1 and combination therapies for targeting multiple signalling pathways are enlisted in Table 2.

**Table 1:** Signalling pathways, key molecules, and therapeutic agents in HCC metastasis.

Pathway	Key Molecules	Therapeutic Agents	Status	References
Wnt/β-Catenin	β-catenin (*CTNNB1*), AXIN1, APC, GSK3β	PRI-724 (CBP/β-catenin inhibitor), LGK974 (Porcupine inhibitor)	Experimental	Craig et al. (2020); Villanueva et al. [235]
TGF-β	TGF-β1, SMAD2/3, SMAD4	Galunisertib (LY2157299), Fresolimumab (anti-TGF-β mAb)	Phase II/Experimental	Giannelli et al. [31]; Neuzillet et al. [236]
PI3K/AKT/mTOR	PTEN, PIK3CA, AKT, mTOR	Everolimus (mTOR inhibitor), MK-2206 (AKT inhibitor)	Approved∗/Experimental	Llovet et al. [12]; Santoro et al. [237]
MAPK/ERK	RAS, BRAF, ERK	Sorafenib, Lenvatinib	Approved (1st-line)	Llovet et al. [116]; Kudo et al. [238]
HGF/c-MET	c-MET, HGF, Gab1	Cabozantinib, Tivantinib	Approved/Phase III	Abou-Alfa et al. [134]; Rimassa et al. [239]
Notch	Notch1/2, HES1, HEY1	RO4929097 (gamma-secretase inhibitor)	Experimental	Takebe et al. [139]
Hippo-YAP/TAZ	YAP, TAZ, MST1/2, LATS1/2	Verteporfin (YAP/TAZ inhibitor)	Preclinical	Liu-Chittenden et al. [240]
Angiogenesis	VEGF, VEGFR2, HIF-1α	Bevacizumab (anti-VEGF), Ramucirumab (anti-VEGFR2)	Approved (combo/2nd-line)	Finn et al. [241]; Zhu et al. [81]
Immune Checkpoints	PD-1, PD-L1, CTLA-4	Nivolumab (anti-PD-1), Atezolizumab (anti-PD-L1) + Bevacizumab	Approved	El-Khoueiry et al. [242]; Finn et al. [241]; Harding, J. J. et al. [243]

**Table 2:** Signalling pathways targeted and combination therapies for HCC.

Therapy	Target/Mechanism	Status (Line)	References
**Sorafenib**	First-line VEGF/RAF pathway inhibitor	FDA-approved (2007, 1L)	[244]
**Lenvatinib**	First-line VEGF/FGF pathway inhibitor	FDA-approved (2018, 1L)	[238]
**Atezolizumab + Bevacizumab**	PD-L1 inhibitor + anti-VEGF monoclonal antibody combination	FDA-approved (2020, 1L)	[241]
**Durvalumab + Tremelimumab**	PD-L1 inhibitor + CTLA-4 inhibitor (STRIDE regimen)	FDA-approved (2022, 1L)	[245,246]
**Nivolumab + Ipilimumab**	PD-1 inhibitor + CTLA-4 inhibitor (ICI combo)	FDA accelerated (2020, 2L)	[247]
**Regorafenib**	Second-line TKI after sorafenib	FDA-approved (2017, 2L)	[244]
**Cabozantinib**	Second-/third-line TKI inhibiting VEGF, MET, AXL	FDA-approved (2019, 2L)	Abou-Alfa et al., 2018
**Ramucirumab**	Second-line (VEGFR2 antibody) monoclonal antibody	FDA-approved (2019, 2L)	[81]
**Tislelizumab**	First-line ICI (PD-1 inhibitor, noninferior to sorafenib in RATIONALE-301 trial)	Phase III (1L, China/Int'l)	[17,248]
**Camrelizumab + Rivoceranib**	PD-1 inhibitor + VEGFR-2 TKI (apatinib) (CARES-310 trial)	Phase III (1L)	[17,248]
**Penpulimab + Anlotinib**	PD-1 inhibitor + VEGFR/FGFR TKI (Phase Ib/II trial)	Phase I/II (1L)	[249]
**MTL-CEBPA (liposomal saRNA)**	Small activating RNA targeting C/EBPα (nano-formulated)	Phase I (1L combo trials)	[250]
**Albumin-bound paclitaxel + Pembrolizumab**	NP-based chemotherapy + PD-1 inhibitor (experimental combination)	Phase I (1L)	[244]
**GPC3-targeted radiopharmaceutical (RYZ801/RYZ811)**	Alpha-emitting targeted therapy (first-in-human trial)	Phase I (investigational)	[244]
**Lipid-nanoparticle siRNA therapies**	Gene-targeted therapies (e.g., DCR-MYC, etc.)	Early-phase trials	[244]

## 5 Conclusion and future perspectives

This review deals with many critical pathways and components involved in HCC metastasis by signalling networks, immune – evasion and remodelling microenvironment. An integrative understanding of these molecular pathways not only elucidates disease mechanisms but also outlines potential therapeutic targets. The clinical relevance of these pathways is especially intriguing, in that they hold the potential to inform the discovery of novel biomarkers for early diagnosis, prognostic assessment, and personalized treatment approaches. In addition, it highlights the need for multidisciplinary management in the optimization of HCC outcomes. Including the various expertise and treatment modalities, healthcare professionals or researchers could create whole patient care plans that are individualized. The approach may include targeted systemic therapies (immunotherapies, TKI/ICI/anti-VEGF), liver directed therapy, surgery/transplant when feasible, and a supportive/palliative care. Such frameworks highlight the critical need for continuing research, translational clinical studies, and interdisciplinary collaborations to optimize treatment paradigms and addressing the limitations for HCC and enhance long-term outcomes for patients.

## Funding

No funding was received to carry out this research.

## CRediT authorship contribution statement

**Jayanta Das:** Conceptualization, Formal analysis, Methodology, Supervision, Writing – original draft, Writing – review & editing. **Bhupen Barman:** Conceptualization, Writing – original draft, Writing – review & editing. **Phulen Sarma:** Conceptualization, Formal analysis, Writing – original draft. **Bipul Kumar Das:** Formal analysis, Methodology, Writing – original draft. **Rajiv Chetia:** Formal analysis, Writing – original draft. **Partha Pratim Kalita:** Conceptualization, Data curation, Formal analysis, Methodology, Resources, Writing – original draft, Writing – review & editing.

## Declaration of competing interest

The author is not an Editorial Board Member/Editor-in-Chief/Associate Editor/Guest Editor for this journal and was not involved in the editorial review or the decision to publish this article.

The authors declare the following financial interests/personal relationships which may be considered as potential competing interests: NONE.

## Data Availability

No data was used for the research described in the article.
